# Preparation of Crosslinked Poly(acrylic acid-*co*-acrylamide)-*Grafted* Deproteinized Natural Rubber/Silica Composites as Coating Materials for Controlled Release of Fertilizer

**DOI:** 10.3390/polym15071770

**Published:** 2023-04-02

**Authors:** Supharat Inphonlek, Kasama Jarukumjorn, Pranee Chumsamrong, Chaiwat Ruksakulpiwat, Yupaporn Ruksakulpiwat

**Affiliations:** 1 School of Polymer Engineering, Institute of Engineering, Suranaree University of Technology, Nakhon Ratchasima 30000, Thailand; 2 Research Center for Biocomposite Materials for Medical Industry and Agricultural and Food Industry, Suranaree University of Technology, Nakhon Ratchasima 30000, Thailand

**Keywords:** modified natural rubber, poly(acrylic acid-*co*-acrylamide), natural rubber composites, controlled release fertilizer, water retention

## Abstract

The crosslinked poly(acrylic acid-*co*-acrylamide)-*grafted* deproteinized natural rubber/silica ((PAA-*co*-PAM)-DPNR/silica) composites were prepared and applied as coating materials for fertilizer in this work. The crosslinked (PAA-*co*-PAM)-DPNR was prepared via emulsion graft copolymerization in the presence of MBA as a crosslinking agent. The modified DPNR was mixed with various contents of silica (10 to 30 phr) to form the composites. The existence of crosslinked (PAA-*co*-PAM) after modification provided a water adsorption ability to DPNR. The swelling degree values of composites were found in the range of 2217.3 ± 182.0 to 8132.3 ± 483.8%. The addition of silica in the composites resulted in an improvement in mechanical properties. The crosslinked (PAA-*co*-PAM)-DPNR with 20 phr of silica increased its compressive strength and compressive modulus by 1.61 and 1.55 times compared to the unloaded silica sample, respectively. There was no breakage of samples after 80% compression strain. Potassium nitrate, a model fertilizer, was loaded into chitosan beads with a loading percentage of 40.55 ± 1.03% and then coated with the modified natural rubber/silica composites. The crosslinked (PAA-*co*-PAM)-DPNR/silica composites as the outer layers had the ability of holding water in their structure and retarded the release of fertilizer. These composites could be promising materials for controlled release and water retention that would have potential for agricultural application.

## 1. Introduction

The controlled release system is an effective strategy for various applications such as biomedical [1,2] and agricultural [3,4] applications that can enable the release of the active ingredients to achieve a desired response. For the agricultural field, water and nutrients are essential for the growth of plants. The fertilizer is the substance that is added to improve agricultural productivity. The fertilizer is usually in a soluble salt form and can dissolve quickly by water. So, it can be leached out from the soil. The leaching of the fertilizer can contaminate natural resources and leads to environmental pollution. Moreover, the addition of a high amount of fertilizer may not be consistent with plant growth and may cause damage to the root system of a plant. Therefore, the design of materials for the coating of fertilizer to allow for controlled release behavior was developed [5,6]. For the controlled release fertilizer, the coating materials should be natural, nontoxic and environmentally friendly materials. They should release nutrients along with the growth rate of plants to support the plant growth. They should retain a lot of water to increase the moisture in the soil and reduce the soil compaction [7]. Therefore, they could improve the physical quality of soil and slow down soil deterioration. Moreover, they should contain functional groups capable of absorbing fertilizer for holding nutrients in the soil and reducing the loss of nutrients.

Bio-based polymeric materials are interesting and have advantages for several fields including agricultural application because of their low cost, low toxicity and environmental friendliness. Natural rubber (NR) is a biopolymer, obtained from *Hevea Brasiliensis* rubber trees. NR has high elasticity and film-forming ability. The excellent properties of NR make it suitable for coating substances [8]. NR can be applied as a film barrier to prevent the release of water-soluble molecules due to its hydrophobic characteristics [9,10]. It contains polyisoprene chains with a low glass transition temperature that is favorable to form rubbery film. However, in order to prepare the natural-rubber-based coating materials with controlled release behavior and water retention ability, the combination and modification with other functional components to have such characteristics are interesting strategies. 

Superabsorbent polymer materials are promising materials for the agricultural sector. They are composed of a three-dimensional polymeric network structure that can absorb and hold water and nutrients during the growth period. Poly(acrylic acid-*co*-polyacrylamide) is a copolymer that possesses hydrophilic properties with a large number of polar groups [11]. J. Zhu et al. successfully prepared poly(acrylic acid-*co*-polyacrylamide)-based superabsorbent materials through the graft copolymerization of okara with acrylic acid and acrylamide [12]. They exhibited an enhanced water retention ability, depending on the contents of the grafted polymer. Their water adsorption capacities were found to be in the range of 120 to 200 g/g in tap water. The enhancement in plant growth was observed, presenting more than 80% determined by the weight and leaf area of the plant. Thus, poly(acrylic acid-*co*-polyacrylamide)-based materials were used as soil supplements for plant growth application under a water-limited condition.

According to the our previous work, poly(acrylic acid-*co*-acrylamide)-*grafted* deproteinized natural rubber ((PAA-*co*-PAM)-DPNR) was successfully prepared via emulsion graft copolymerization which was a water-based process [13]. This system was environmentally friendly and safe without using organic solvents. The PAA-*co*-PAM was grafted on the particle surface of natural rubber. The monomer contents were varied at 10 and 30 phr. The grafting efficiency and grafting percentage were found in the range of 20.8–38.9 and 2.1–9.9%, respectively. However, when the products were used in the wet condition, the ungrafted polymers could be dissolved in the aqueous medium. So, the undesired performance in application was achieved. Moreover, the modified natural rubber was then mixed with silica. The modified natural rubber/silica composites showed enhanced mechanical properties due to strong interactions between the silica and modified natural rubber. Natural rubber/filler composites have been studied for improving the properties of natural-rubber-based materials, including mechanical and thermal properties to promote application efficacy meaning they can be used in a wide range of applications. The silica-reinforced natural rubber composites were used for many rubber applications such as tire manufacturing [14]. The composites can be used as the supporting frameworks for the adsorption process [15]. N.C. Oliveira et al. prepared the composites of natural rubber/fluorescent silica particles [16]. The composites exhibited good structural stability under fluid flow and high ionic strength conditions (NaCl, 0.85% *w/v*). Therefore, the natural rubber/silica-based materials could be applied for use in various coating applications such as biomedical devices, microelectronics and the automobile industries. P. Boonying et al. prepared the biocomposite coating films from lignin and natural rubber grafted with polyacrylamide (NR-*g*-PAM) for slow release fertilizer [17,18]. The lignin/NR composites used as the outer coating shell of fertilizer can enhance mechanical resistance from the osmotic pressure building up. NR-*g*-PAM acts as the compatibilizer to enhance the stability of lignin/NR dispersions. It can enhance the hydrophilicity to the composites and allow water transport through the lignin/NR composite film. The Li/NR-*g*-PAM-coated urea revealed a slow release of N, but only 60% of the total N after 112 days. Therefore, the natural rubber/filler composites could have potential for various applications.

In this work, to prepare the natural-rubber-based composites for coating fertilizer, the deproteinized natural rubber was modified via emulsion graft copolymerization with acrylic acid and acrylamide. In order to enhance the grafting of PAA-*co*-PAM on the DPNR, the crosslinked (PAA-*co*-PAM)-DPNR was prepared using N’,N’-Methylenebisacrylamide (MBA) as a crosslinking agent. The crosslinked (PAA-*co*-PAM)-DPNR latex was then mixed with silica to prepare the natural rubber/silica composites and applied as coating materials for fertilizer. The crosslinked (PAA-*co*-PAM)-DPNR/silica composites were characterized and coated on chitosan beads. The release behavior and water retention ability of the crosslinked (PAA-*co*-PAM)-DPNR/silica-covered chitosan beads were investigated. 

## 2. Materials and Methods

### 2.1. Materials

Natural rubber latex preserved with high ammonia (NR; 60% of dry rubber content) was purchased from Chemical and Materials Co., Ltd. (Bangkok, Thailand). Acrylic acid (AA) and cumene hydroperoxide (CHP) were obtained from Aldrich (St. Louis, MO, USA). The purification of the AA monomer was performed by passing through a column packed with alumina adsorbent before polymerization [19]. Acrylamide (AM) monomer was obtained from Loba Chemie Pvt. Ltd. (Mumbai, India). Tetraethylene pentamine (TEPA) and chitosan (CS, molecular weight of 100,000–300,000 g/mol) were purchased from Acros organics (Geel, Belgium). Terric16A (10% w) was obtained from Rubber Authority of Thailand (Bangkok, Thailand). N’,N’-Methylenebisacrylamide (MBA) and sodium tripolyphosphate (TPP) were purchased from Alfa Aesar (Haverhill, MA, USA). Silica was prepared via the precipitation method from rice husk ash (byproduct from biomass power plants from Chia Ment Co., Ltd., Nakhon Ratchasima, Thailand) with an average size of 44.02 ± 5.06 nm, determined via SEM. Potassium nitrate (KNO_3_) was obtained from Kemaus (New South Wales, Australia). Deionized (DI) water was used throughout the study. 

### 2.2. Preparation of Crosslinked Poly(acrylic acid-co-acrylamide)-Grafted Deproteinized Natural Rubber

The crosslinked (PAA-co-PAM)-DPNR via emulsion graft copolymerization was performed according to a previous study with some modifications [13]. The deproteinized natural rubber (DPNR) latex was prepared via treatment with urea in the presence of sodium dodecyl sulfate [20]. The DPNR latex and 5 phr of Terric16A were added into the three-necked reactor and stirred at 100 rpm using a mechanical stirrer under nitrogen atmosphere for 45 min. After that, the chemicals were injected into the reactor as follows: CHP, acrylic acid (40 mol% of acrylic acid was neutralized with 20% w of NaOH solution), acrylamide, MBA and TEPA. The CHP and TEPA were fixed as 1 phr. The content of the comonomer was used at 30 phr with 50:50 by weight ratio of acrylic acid and acrylamide. The MBA contents were varied as 0.25 and 0.50% w of monomer. The total solid content was kept constant at 15 wt%. The polymerization was carried out at 50 °C for 6 h. 

### 2.3. Characterization of Crosslinked (PAA-co-PAM)-DPNR 

#### 2.3.1. Determination of Monomer Conversion, Grafting Efficiency and Grafting Percentage

To determine the monomer conversion, the dried samples were weighed before and after immersion in ethanol for 24 h. The samples were then dried at 60 °C for 24 h. The monomer conversion was calculated as follows [21]:

(1) $$\mathrm{Monomer}\;\mathrm{conversion}\;\left(\%\right)=\frac{\mathrm{Weight}\;\mathrm{of}\;\mathrm{polymer}\;\mathrm{formed}}{\mathrm{Weight}\;\mathrm{of}\;\mathrm{total}\;\mathrm{monomer}\;\mathrm{added}}\times 100.$$

For the calculation of grafting efficiency and grafting percentage, the ungrafted PAA-*co*-PAM was removed via extraction with DI water. The dried samples were immersed with DI water for 72 h. The medium was changed every 8 h. The samples were then dried at 60 °C for 24 h. The grafting efficiency and grafting percentage were calculated as follows [22]:

(2) $$\mathrm{Grafting}\;\mathrm{efficiency}\;\left(\%\right)=\frac{\mathrm{Weight}\;\mathrm{of}\;\left(\mathrm{PAA}\;-co-\;\mathrm{PAM}\right)\;\mathrm{grafted}}{\mathrm{Weight}\;\mathrm{of}\;\mathrm{total}\;\mathrm{polymer}\;\mathrm{formed}}\times 100$$

(3) $$\mathrm{Grafting}\;\mathrm{percentage}\;\left(\%\right)=\frac{\mathrm{Weight}\;\mathrm{of}\;\left(\mathrm{PAA}\;-co-\;\mathrm{PAM}\right)\;\mathrm{grafted}}{\mathrm{Weight}\;\mathrm{of}\;\mathrm{DPNR}\;\mathrm{used}}\times 100.$$

#### 2.3.2. Morphology

The morphology of samples was studied using transmission electron microscopy (TEM). A drop of diluted latex was put onto a carbon-coated copper grid. The sample was stained with osmium tetroxide in the carbon–carbon double bonds of natural rubber for 24 h to increase the contrast [23]. The morphology was observed through a transmission electron microscope using Talos F200X (Thermo Fisher Scientific, Waltham, MA, USA) at 120 kV.

#### 2.3.3. Gel Fraction

The dried samples were weighed and then immersed in DI water to extract the sol fraction from the matrix at room temperature for 72 h. The medium was changed 3 times a day. The samples were subsequently freeze-dried overnight. The samples were weighed after drying and the gel fraction was calculated as follows [24]:

(4) $$\mathrm{Gel}\;\mathrm{fraction}\;\left(\%\right)=\;\frac{\mathrm{Wf}}{\mathrm{Wi}}\times 100$$

where Wi is the initial weight of dried samples and Wf is the weight of dried samples after immersion in water and freeze drying. 

### 2.4. Preparation of Crosslinked (PAA-co-PAM)-DPNR/Silica Composites

The crosslinked (PAA-*co*-PAM)-DPNR/silica composites were prepared by mixing the crosslinked (PAA-*co*-PAM)-DPNR latex with different silica contents. The silica was prepared via the precipitation method by treatment with rice husk ash with 1 M of HCl solution for 3 h to remove the metallic oxide. The ash was then filtered and washed with DI water until the pH was neutral. The purified ash was dried in the hot air oven at 110 °C for 12 h. The dried sample was added into 1 M of NaOH solution and stirred for 12 h at 90 °C to obtain the sodium silicate. The undissolved product was removed via filtration. To prepare the silica, 1 M of acetic acid was dropped into the sodium silicate solution until the pH was neutral under stirring at room temperature. The precipitate silica was obtained and then separated via filtration followed by washing with the excess of DI water. The resulting silica was dispersed in DI water with the solid content of 3%. The silica dispersion was dropped into the latex and stirred at 100 rpm for 3 h at room temperature. The silica contents were varied as 10, 20 and 30 phr. The chemical compositions are shown at Table 1. The mixture was poured into the plastic mold and dried at 60 °C for 24 h. The samples were kept for further characterization. 

### 2.5. Characterization of Crosslinked (PAA-co-PAM)-DPNR/Silica Composites

#### 2.5.1. Fourier-Transform Infrared Spectroscopy

Fourier-transform infrared spectroscopy (FTIR) with an attenuated total reflection (ATR) mode was used to study the chemical structure of the crosslinked (PAA-*co*-PAM)-DPNR/silica composites using a Tensor 27 FTIR spectrometer (Bruker, Billerica, MA, USA). The scanning of each spectrum was performed with 64 scans at a resolution of 4 cm^−1^. The FTIR spectra of all samples were recorded in the range of 4000–400 cm^−1^.

#### 2.5.2. Morphology

The morphology of samples was observed using a scanning electron microscope (SEM). The samples were frozen and broken in liquid nitrogen. The samples were fixed on the stub using conductive carbon tape and kept in a desiccator overnight. The samples were sputter-coated with gold under a vacuum for 3 min. The cross-section of samples was observed through a JSM-6010LV (JEOL, Tokyo, Japan). Moreover, the element compositions of the crosslinked (PAA-*co*-PAM)-DPNR/silica composites were determined via energy-dispersive spectroscopy coupled with SEM (SEM/EDS).

#### 2.5.3. Swelling Degree

To determine the water absorption ability of samples, the swelling test was carried out via immersion in DI water for 24 h. The swollen samples were taken out from the medium and wiped with filter paper. Then, the swollen samples were weighed. The swelling degree was calculated as follows [25]:

(5) $$\mathrm{Swelling}\;\mathrm{degree}\;\left(\%\right)=\frac{\mathrm{Ws}-\mathrm{Wi}}{\mathrm{Wi}}\times 100$$

where Wi is the initial weight of dried samples and Ws is the weight of swollen samples.

#### 2.5.4. Contact Angle

The contact angle measurement was carried out by dropping water via a microsyringe on the rubber sample. After 20 s, the water droplet on the surface was recorded and the angle formed between the interface was measured using ImageJ software (IJ 1.46r image analyzer software).

#### 2.5.5. Thermogravimetric Analysis

The thermal properties of the composites were traced via thermogravimetric analysis using a TGA/DSC1 (Mettler Toledo, Columbus, OH, USA). A total of 10 mg of the dried sample was added in a sample pan. The pan without the addition of the sample was used as a reference. The reference and sample pans were placed into the furnace. The measurement was performed in the temperature range of 50 to 600 °C at a heating rate of 10 °C/min under nitrogen atmosphere.

#### 2.5.6. Compressive Properties

The compressive properties of the crosslinked (PAA-*co*-PAM)-DPNR/silica composites were determined using a TA.XT plus texture analyzer (Stable Micro systems Ltd., Surrey, UK). The sample was immersed in DI water before measurement and then cut into a cylindrical shape with a diameter of approximately 10 mm and a thickness of 2 mm. The swollen sample was compressed to 80% strain with a fixed strain rate at 0.05 mm/s at room temperature. The compressive modulus was determined from the slope of the stress–strain curve (5–10% strain) [26]. The measurement was repeated six times for each sample.

### 2.6. Preparation and Characterization of Crosslinked (PAA-co-PAM)-DPNR/Silica-coated CS Beads

#### 2.6.1. Preparation

The chitosan beads were prepared following the ionic gelation technique according to J.J. Perez et al. [27]. The 10 g of 3% *w*/*w* of chitosan (CS) solution in acetic acid (1% *v*/*v*) was dropped into 50 mL of sodium tripolyphosphate (TPP) solution using a syringe with a needle. The concentration of TPP was kept constant at 1% *w*/*v*. The sample was continuously stirred at 200 rpm for 4 h. The CS beads were removed from the solution and washed with DI water. The beads were then dried at 35 °C for 24 h. The crosslinked (PAA-*co*-PAM)-DPNR/silica latex with various silica contents was dropped on CS beads. The weight ratio of crosslinked (PAA-*co*-PAM)-DPNR/silica and CS was kept constant at 1:15. The sample was dried at 60 °C for 24 h to obtain crosslinked (PAA-*co*-PAM)-DPNR/silica-coated CS beads.

#### 2.6.2. Morphology

The morphology of the prepared beads was observed using a scanning electron microscope (SEM). Before SEM observation, the crosslinked (PAA-*co*-PAM)-DPNR/silica-coated CS beads were immersed in DI water for 1 h. The samples were subsequently freeze-dried overnight. The freeze-dried beads were fixed on a stub using conductive carbon tape. Then, they were sputter-coated with gold under vacuum for 3 min. The samples were subjected to FEI Quanta 450 SEM (Philips, Hillsboro, OR, USA).

#### 2.6.3. Water Retention

The water retention test was performed by measuring the change in the remaining weight of water in the sample container. The sample container was filled with the sample and sand (Wo). Sand with a size in the range of 425–625 µm determined via passing it through a sieve mesh was dried at 105 °C for 24 h before testing. The different types of beads (5% w) were buried in 30 g of sand in a plastic cup, followed by the addition of DI water (30% w) (Ws). The weight of the sample container was recorded at certain time intervals (Wt). The measurement was performed at 25 and 45 °C. The water retention was calculated as follows [28,29]:

(6) $$\mathrm{Water}\;\mathrm{retention}\;\left(\%\right)=\frac{\mathrm{Wt}\;-\;\mathrm{Wo}}{\mathrm{Ws}\;-\;\mathrm{Wo}}\times 100.$$

#### 2.6.4. Loading Percentage

To encapsulate potassium nitrate in the CS beads, the dried CS beads were immersed in 20% w of potassium nitrate solution for 4 h. Subsequently, the samples were dried at 35 °C for 24 h [27]. The loading percentage was calculated according to the following equation:

(7) $$\mathrm{Loading}\;\mathrm{percentage}\;\left(\%\right)=\frac{\mathrm{Wa}\;-\;\mathrm{Wb}}{\mathrm{Wb}}\times 100$$

where Wa is the weight of the dried sample after loading and Wb is the initial weight of dried beads, respectively.

#### 2.6.5. Release Behavior

The release experiment was performed by placing potassium-nitrate-loaded beads in a dialysis bag (CelluSep T4, molecular cut-off 6–8 kDa) and then immersed in 100 mL of DI water. The test was performed at 25 °C. The released amount of potassium nitrate was determined by measuring the conductivity of aqueous medium at various time intervals.

## 3. Results and Discussion

### 3.1. Preparation and Characterization of Crosslinked (PAA-co-PAM)-Grafted Deproteinized Natural Rubber

#### 3.1.1. Conversion, Grafting Efficiency and Grafting Percentage

**Figure 1:**
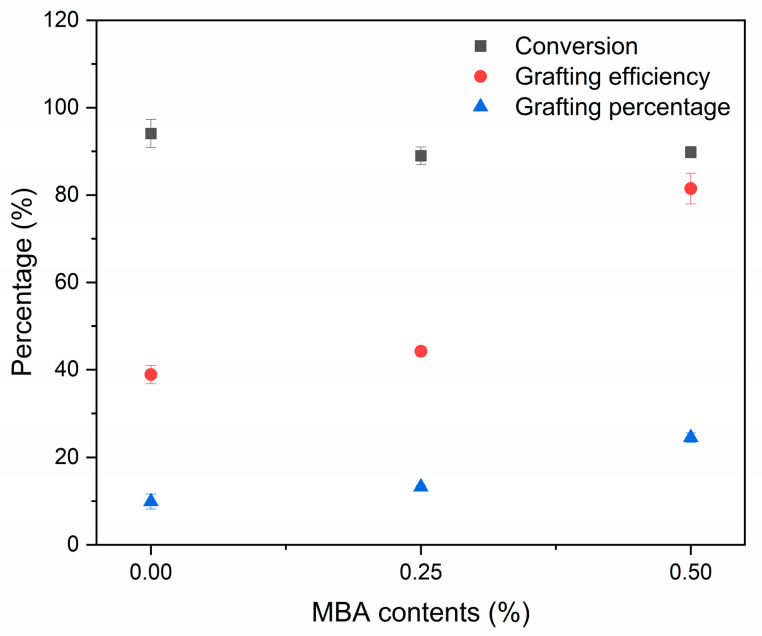
Effect of crosslinking agent contents on the monomer conversion, grafting efficiency and grafting percentage.

The MBA-crosslinked (PAA-*co*-PAM)-DPNR was prepared via graft copolymerization of a comonomer of acrylic acid and acrylamide. The MBA crosslinking agent was introduced during the polymerization process and its contents were varied at 0.25 and 0.50 by weight percentage of the comonomer, which were noted as M0.25/P30-DPNR and M0.50/P30-DPNR, respectively. The added crosslinking agent can link the PAA-*co*-PAM chains and form a crosslinked network of hydrophilic polymers into the natural-rubber-based matrix as the potential materials for application [30]. The properties of the crosslinked (PAA-*co*-PAM)-DPNR with different MBA contents were compared to the uncrosslinked sample (P30-DPNR). From the results shown in Figure 1, it was found that the conversion was found in the range of 89.0 ± 2.0 to 94.1 ± 3.2%. When the crosslinking agent was added, the grafting efficiency and grafting percentage were increased as compared to P30-DPNR. The grafting efficiency increased from 38.9 ± 2.1 to 81.5 ± 3.5%, and the grafting percentage increased from 9.9 ± 1.7 to 24.4 ± 1.1% when the crosslinking agent was increased from 0 to 0.50% w of the comonomer. The addition of the crosslinking agent allows for interactions between the PAA-*co*-PAM molecular chains and can link the ungrafted polymers to the natural-rubber-based structure. This resulted in an increase in the grafting efficiency and grafting percentage of the crosslinked samples.

#### 3.1.2. Morphology

**Figure 2:**
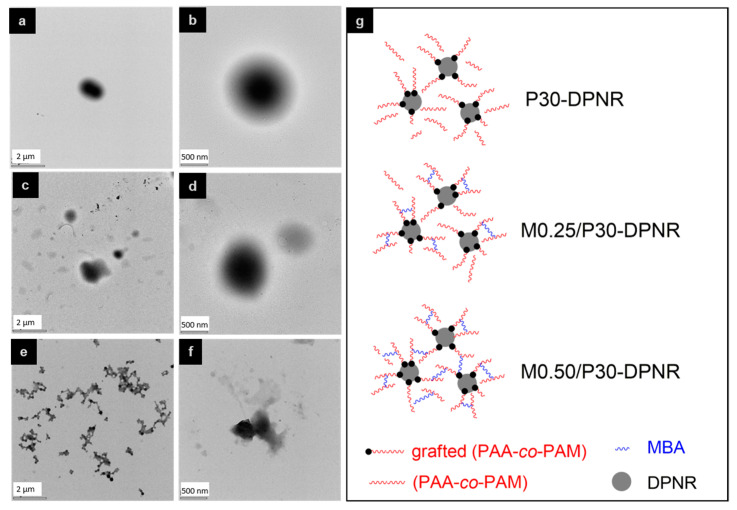
TEM images of (**a**,**b**) P30-DPNR, (**c**,**d**) M0.25/P30-DPNR and (**e**,**f**) M0.50/P30-DPNR, and (**g**) schematic representation of the formation of crosslinked (PAA-*co*-PAM)-DPNR.

Figure 2 shows the morphology of the uncrosslinked and crosslinked (PAA-*co*-PAM)-DPNR particles. From the TEM images, the particles exhibited a core–shell morphology. All particles showed the dark color particles of DPNR covered with the (PAA-*co*-PAM) shell [31]. This confirmed the presence of PAA-*co*-PAM after modification. After adding the MBA crosslinking agent, it could be clearly seen that at 0.50% w of MBA, the formation of linkage around the rubber surface occurred, suggesting that the network structure took place between rubber particles [32]. This can be explained by the reaction mechanism for preparing the grafted natural rubber using the CHP/TEPA system. According to D.J. Lamb et al. [33], the free radicals were generated on the natural rubber chains. The graft copolymerization was then performed on the rubber surface to form the polymer grafted natural rubber. However, in this approach, the ungrafted polymer also occurred from the chain transfer reaction. Therefore, in the condition without the addition of a crosslinking agent, the ungrafted PAA-*co*-PAM can be formed and covered on the natural rubber surface without being linked by chemical bonds, as seen in Figure 2g. When the MBA was added, it reacted with the formed PAA-*co*-PAM during the chain propagation [34]. Therefore, the crosslink between the grafted PAA-*co*-PAM chains could have resulted from and also be associated with ungrafted chains. Nevertheless, the addition of a high amount of crosslinking agent not only chemically bonded with PAA-*co*-PAM chains on the individual natural rubber particles, but it might have also led to crosslinking them on the other rubber particles.

#### 3.1.3. Gel Fraction

**Figure 3:**
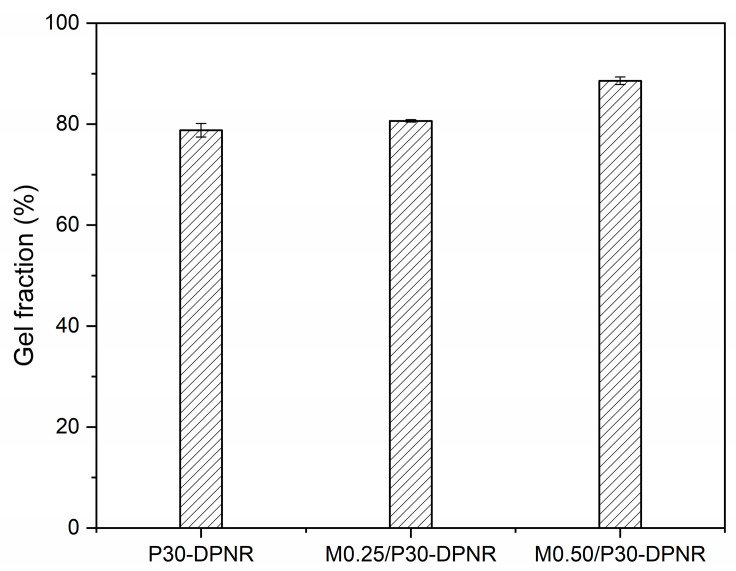
Gel fraction of P30-DPNR, M0.25/P30-DPNR and M0.50/P30-DPNR.

The gel fraction of the modified natural rubber was studied to investigate the ability of samples for holding the (PAA-*co*-PAM) chains in their structure. From Figure 3, it was observed that the gel fraction of crosslinked (PAA-*co*-PAM)-DPNR was higher than that of the uncrosslinked sample. The gel fraction of P30-DPNR was found to be 78.8 ± 1.3%. When the MBA crosslinking agent was introduced, the gel fraction increased to 80.6 ± 0.2 and 88.6 ± 0.8% for M0.25/P30-DPNR and M0.50/P30-DPNR, respectively. It can be seen that the addition of a higher crosslinking agent leads to an increase in the gel fraction due to a larger crosslinking site in the sample. Indeed, natural rubber is a hydrophobic molecule composed of polyisoprene chains, while the PAA-*co*-PAM is a hydrophilic polymer. When the samples are immersed in aqueous medium, the ungrafted PAA-*co*-PAM segments can dissolve in aqueous medium, resulting in a reduction in the gel fraction. The crosslinking agent interacted with PAA-*co*-PAM chains and linked them to a rubber-based structure via chemical bonds. Thus, it was suggested that the presence of an MBA crosslinking agent can improve structural stability and hold the hydrophilic units on the sample structure. This would bring advantages for use in application in terms of water adsorption ability [35,36].

### 3.2. Preparation and Characterization of Crosslinked (PAA-co-PAM)-DPNR/Silica Composites

#### 3.2.1. Preparation of Crosslinked (PAA-*co*-PAM)-DPNR/Silica Composites

**Figure 4:**
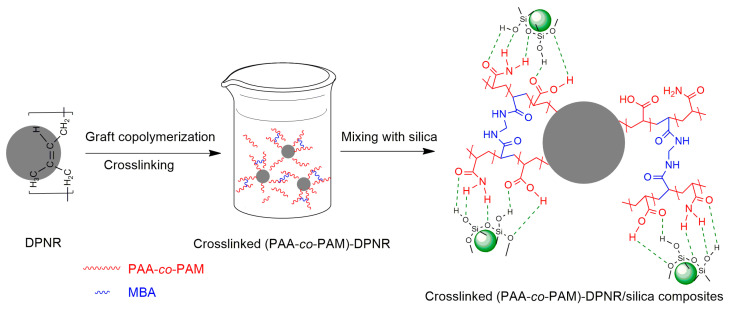
Schematic representation of the fabrication of the crosslinked (PAA-co-PAM)-DPNR/silica composites.

The crosslinked (PAA-*co*-PAM)-*grafted* deproteinized natural rubber/silica composites were prepared to be used as coating materials for fertilizer. From the above section, the crosslinked (PAA-*co*-PAM)-*grafted* DPNR was firstly prepared via emulsion graft copolymerization using the CHP/TEPA redox initiator system in the presence of MBA as a crosslinking agent. The crosslinked (PAA-*co*-PAM)-DPNR latex was then mixed with silica via the wet mixing process to produce natural rubber/silica composites, as shown in Figure 4. According to the results from Section 3.1, the M0.50/P30-DPNR with the highest grafting efficiency, grafting percentage and gel fraction was employed to be mixed with different silica contents at 10, 20 and 30 phr for preparing the composites, which were named as M0.50/P30-DPNR/Si10, M0.50/P30-DPNR/Si20 and M0.50/P30-DPNR/Si30, respectively.

#### 3.2.2. FTIR Analysis

**Figure 5:**
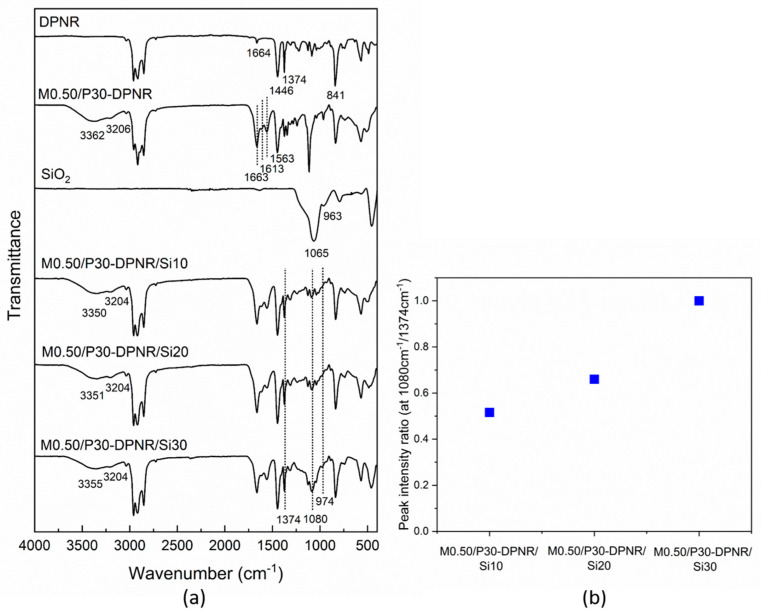
(**a**) FTIR of the DPNR, M0.50/P30-DPNR, silica and M0.50/P30-DPNR/silica composites prepared using different silica contents; (**b**) the relationship of peak intensity ratio at 1080 cm^−1^/1374 cm^−1^ of the crosslinked (PAA-co-PAM)-DPNR/silica composites.

The FTIR was used to characterize the chemical structure and functional groups of the prepared samples, as shown in Figure 5a. The FTIR spectra of the crosslinked (PAA-*co*-PAM)-DPNR/silica composites were compared to DPNR, M0.50/P30-DPNR and silica. The characteristic peaks of DPNR appeared at 1664, 1446, 1374 and 841 cm^−1^, which were assigned to the vibration bands of C=C, -CH_2_, -CH_3_ and =CH, respectively [37]. The M0.50/P30-DPNR shows additional peaks compared to DPNR. Peaks at 3362 cm^−1^ (OH stretching), 3206 cm^−1^ (NH stretching), 1663 cm^−1^ (C=O stretching), 1613 cm^−1^ (NH bending), 1563 cm^−1^ (-COO^−^) and 1240 cm^−1^ (C-O stretching) were observed [38]. These correspond to polyacrylic acid and polyacrylamide, indicating the successful graft copolymerization. For the silica, its spectrum shows the peaks at 1065 cm^−1^ (Si-O-Si asymmetric stretching), 963 cm^−1^ (Si-OH bending), 794 cm^−1^ (Si-O symmetric stretching) and 458 cm^−1^ (Si-O bending) [39]. In the case of the crosslinked (PAA-*co*-PAM)-DPNR/silica composites, both characteristic peaks of M0.50/P30-DPNR and silica appeared. However, the peaks of OH stretching and NH stretching were shifted to 3350 cm^−1^ and 3204 cm^−1^, respectively. The peaks of Si-OH and Si-O-Si were shifted from 963 to 974 cm^−1^ and 1065 to 1080 cm^−1^, respectively. These demonstrated the generation of H-bonding between modified natural rubber and silica and confirmed the incorporation of silica in the composites [40]. Moreover, the peak intensity ratios of the composites calculated by the peak intensity at 1080 cm^−1^ of Si-O-Si stretching compared to those at 1374 cm^−1^ of -CH_3_ stretching are shown in Figure 5b. It was found that the intensity ratios increased when the silica contents increased. The increase in the ratio corresponds to the increasing amount of silica added.

#### 3.2.3. Morphology

**Figure 6:**
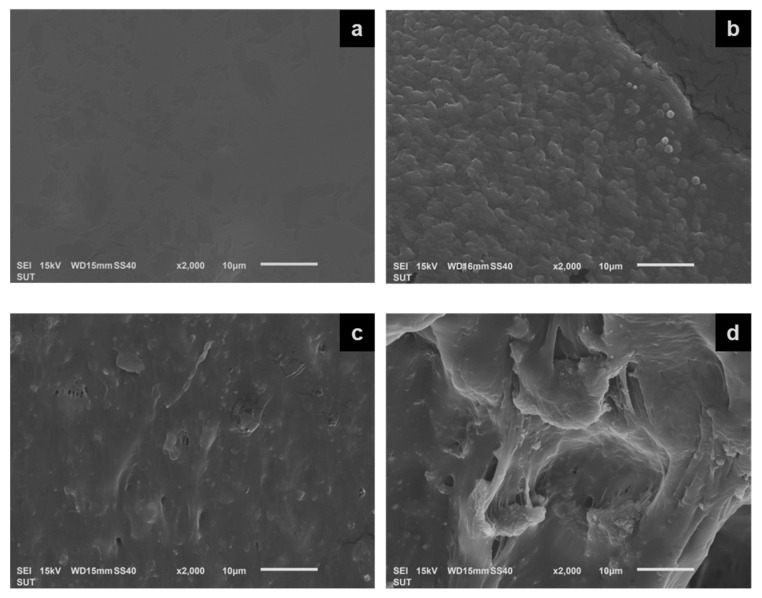
SEM images of cross-section surface of (**a**) M0.50/P30-DPNR, (**b**) M0.50/P30-DPNR/Si10, (**c**) M0.50/P30-DPNR/Si20 and (**d**) M0.50/P30-DPNR/Si30.

Figure 6 presents the SEM images of the crosslinked (PAA-*co*-PAM)-DPNR/silica composites compared to the sample without the addition of silica. As seen in Figure 6a, the M0.50/P30-DPNR had a smooth surface. When the silica was introduced at 10, 20 and 30 phr, the cross-section surface of the M0.50/P30-DPNR/silica composites (Figure 6b–d) exhibited a rough surface by the presence of silica particles dispersed in the rubber matrix. The silica particles had a size of about 44.02 ± 5.06 nm, as observed in Figure S1 (see supplemental information). When the silica was added into the system at 10 phr, the good dispersion of silica particles was obtained. The strong interaction between the polar functional groups of modified DPNR and silica resulted in the formation of rubber–silica clusters with a size of about 1.62 ± 0.27 µm, which was larger than that of silica particles. When the amount of silica was increased, more interaction was obtained, resulting in an uneven layer as observed from SEM images. However, when the silica was increased up to 30 phr, the large pit and the agglomeration of silica particles were found.

#### 3.2.4. Swelling Degree

**Figure 7:**
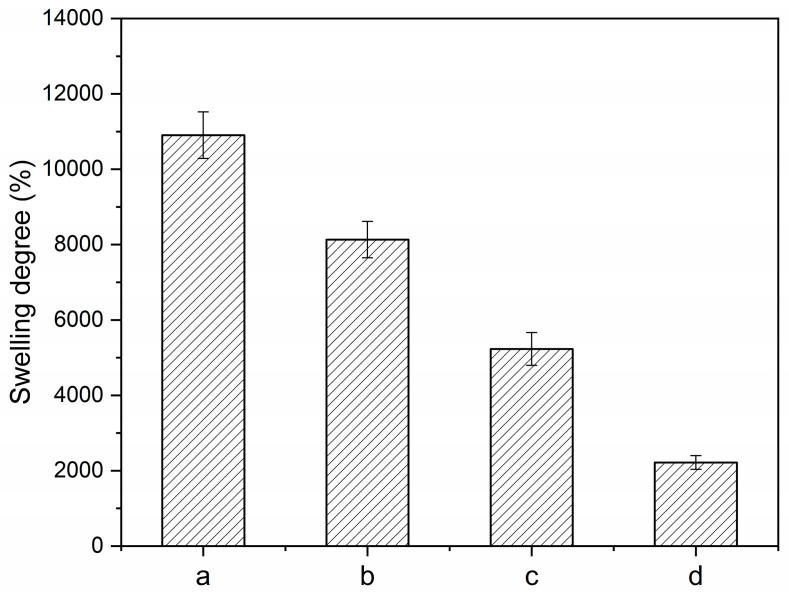
Swelling degree of (**a**) M0.50/P30-DPNR, (**b**) M0.50/P30-DPNR/Si10, (**c**) M0.50/P30-DPNR/Si20 and (**d**) M0.50/P30-DPNR/Si30 after immersion in DI water for 24 h.

The swelling degree and characteristics of the composites after immersion in water were determined to estimate the water absorption capacity of the composites, as displayed in Figure 7. The M0.50/P30-DPNR film gradually expands in the medium as it can absorb water into its structure. The swelling degree of M0.50/P30-DPNR was found as 10,905.5 ± 617.9%, while no dimensional change was observed for DPNR when immersed in water due to its hydrophobic characteristic. The swelling degree of DPNR was found to only be 4.3 ± 0.5%. Thus, the existence of crosslinked PAA-*co*-PAM in natural-rubber-based materials can promote the water adsorption capacity due to crosslink network structure formation, together with polar functional groups such as carboxylic acid and amide groups presenting in the chemical structure of PAA-*co*-PAM for the highly effective absorption. When the silica was added, the composites showed a lower swelling degree than that of MBA0.50/P30-DPNR. The swelling degree of M0.50/P30-DPNR/Si10, M0.50/P30-DPNR/Si20 and M0.50/P30-DPNR/Si30 were 8132.3 ± 483.8, 5232.1 ± 435.6 and 2217.3 ± 182.0%, respectively. The swelling degree decreased when silica increased. This could be explained by the fact that since the polar functional groups of PAA-*co*-PAM play an important role in the adsorption process, these groups are strongly interacted with silica and obtain more compact structures. This might lead to an increase in the crosslink density in the composites. A higher crosslink density results in a lower swelling degree, as can be observed in other research works [41]. In addition, the disintegration of all samples in aqueous medium was not found. They showed good physical stability and water adsorption ability that would be useful in application.

#### 3.2.5. Contact Angle

**Figure 8:**
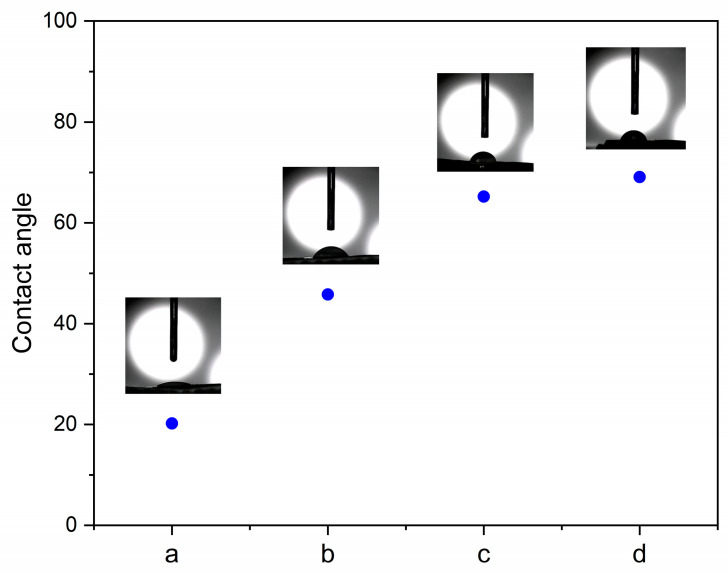
Contact angle of (**a**) M0.50/P30-DPNR, (**b**) M0.50/P30-DPNR/Si10, (**c**) M0.50/P30-DPNR/Si20 and (**d**) M0.50/P30-DPNR/Si30.

Figure 8 presents the contact angle of the M0.50/P30-DPNR and M0.50/P30-DPNR/silica composites with various silica contents. The contact angle of DPNR was reported in the literature to be about 96.0° [42]. As can be seen from the result, the contact angle of M0.50/P30-DPNR was 20.2°, which was lower than that of DPNR. The decrease in the contact angle indicated that the M0.50/P30-DPNR was more hydrophilic compared to DPNR. It is suggested that the presence of PAA-*co*-PAM after modification can enhance the hydrophilicity to the natural-rubber-based structure. For the M0.50/P30-DPNR/silica composites, their contact angle values were higher than those of M0.50/P30-DPNR and tended to increase with an increase in silica contents. The contact angle values of M0.50/P30-DPNR/Si10, M0.50/P30-DPNR/Si20 and M0.50/P30-DPNR/Si30 were found to be 45.8°, 65.2° and 69.1°, respectively. These results were corresponded to the swelling experiment. The composites with more interaction between polar groups of PAA-*co*-PAM and silica were more hydrophobic and showed a lower swelling degree. However, their contact angle values were lower than that of DPNR, suggesting that they still had hydrophilic behavior in their structure.

#### 3.2.6. Thermal Properties

**Figure 9:**
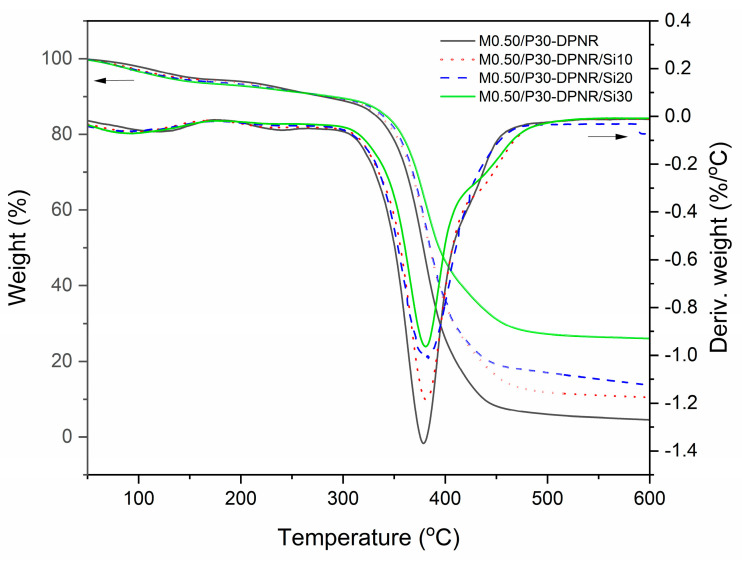
TGA and DTG thermograms of M0.50/P30-DPNR and M0.50/P30-DPNR/silica composites with various silica contents.

The TGA and DTG thermograms of M0.50/P30-DPNR and M0.50/P30-DPNR/silica composites with various silica contents are displayed in Figure 9. The decomposition of samples in the temperature range of 50 to 600 °C were determined. The decomposition at 70–175 °C was found due to the loss of water in the samples. The weight loss between 175–290 °C corresponded to the decomposition of the carboxylic acid and amide side groups of the PAA-*co*-PAM chains [43]. The DPNR and polymer backbone of PAA-*co*-PAM decomposed at a temperature between 336 and 475 °C. From thermograms, the temperature at 10% of weight loss (T_10_), temperature at maximum process rate (T_max_) and residue of the degradation process are reported in Table 2. It was observed that the presence of silica caused the increase in the decomposition temperature of the composites. Their T_10_ and T_max_ values increased when the silica contents increased. Similar results have been reported in other research works [44]. The shift of T_10_ and T_max_ to a higher temperature demonstrated that the composites had stability over wide temperature ranges. It could be indicated that the thermal stability of the composites was improved because the silica particles dispersed in the natural rubber matrix could adsorb heat energy and retard the heat transfer to natural rubber [45]. Moreover, the residues of M0.50/P30-DPNR/silica composites with 10, 20 and 30 phr of silica were found at 10.47, 13.68 and 26.03%, respectively. The results showed that their residues increased with increasing the silica contents and that they were higher than those of M0.50/P30-DPNR (4.52%) due to the higher content of silica in the composites.

#### 3.2.7. Compressive Properties

**Figure 10:**
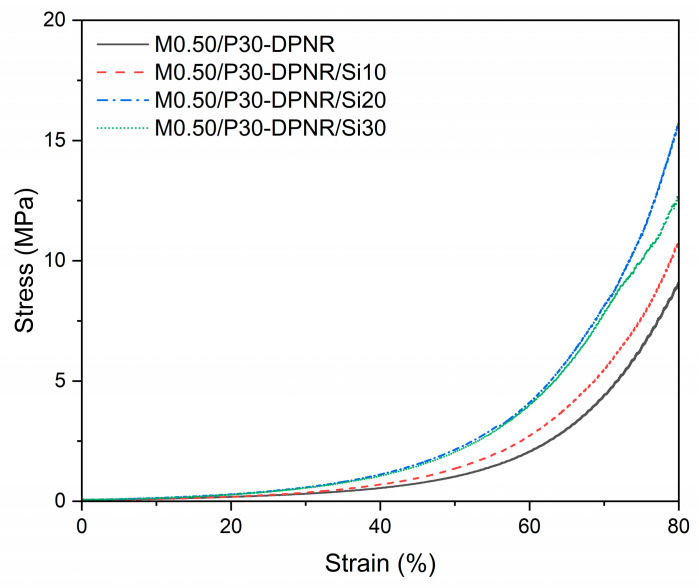
Stress–strain curve of the M0.50/P30-DPNR, M0.50/P30-DPNR/Si10, M0.50/P30-DPNR/Si20 and M0.50/P30-DPNR/Si30 composites.

The mechanical properties of the composites are also important characteristics for the application. Since composites are used as coating materials for fertilizer in agricultural applications, they might be soaked in water and buried in the soil. Therefore, the compressive properties of the composites were determined in the condition of swollen samples with water. Figure 10 displays the stress–strain curve of M0.50/P30-DPNR and M0.50/P30-DPNR/silica composites with different silica contents. The measurement was performed under compression mode and 0 to 80% strain. From the stress–strain curve, it was found that the stress increased with the increasing of strain. The compressive strength at 80% strain and compressive modulus are summarized in Table 3. For M0.50/P30-DPNR, the compressive strength and compressive modulus were 10.71 and 0.71 MPa, respectively. In the case of M0.50/P30-DPNR/silica composites, their compressive strength and compressive modulus were higher than those of M0.50/P30-DPNR and seemed to increase with an increase in silica contents. When the silica was varied from 0 to 20 phr, the compressive strength increased from 10.71 ± 0.94 to 17.29 ± 1.99 MPa and the compressive modulus increased from 0.71 ± 0.13 to 1.10 ± 0.13 MPa. The enhancement in the compressive strength and compressive modulus was obtained by the addition of silica particles. This was because of the replacement of the rubber matrix with the rigid silica particles. Furthermore, when the silica was increased, a stronger interaction between modified DPNR and silica resulted, leading to an improvement in mechanical properties. However, when the silica was added to 30 phr, the compressive strength and compressive modulus of the composites were decreased to 14.27 ± 1.64 and 0.98 ± 0.23 MPa, respectively. This was probably because the addition of large amounts of silica caused the agglomeration of silica, resulting in undesired mechanical properties. Therefore, M0.50/P30-DPNR/Si20 showed the highest compressive strength and compressive modulus, which increased by 1.61 and 1.55 times compared to M0.50/P30-DPNR, respectively. In addition, all samples can maintain their structures without the breakage of samples after increasing the compression strain up to 80%. Thus, these composites would be useful for application.

### 3.3. Fabrication of Crosslinked (PAA-co-PAM)-DPNR/Silica-Coated CS Beads

#### 3.3.1. Morphology

**Figure 11:**
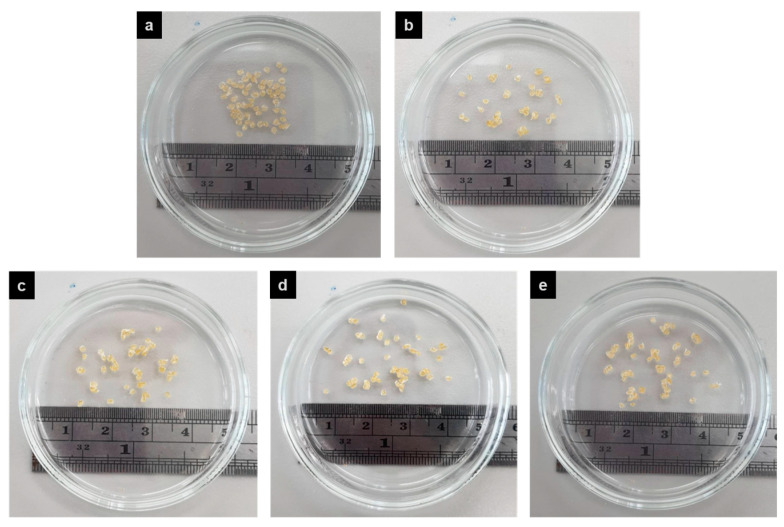
Physical appearance of (**a**) CS beads; (**b**) M0.50/P30-DPNR-coated CS beads; (**c**) M0.50/P30-DPNR/Si10-coated CS beads; (**d**) M0.50/P30-DPNR/Si20-coated CS beads and (**e**) M0.50/P30-DPNR/Si30-coated CS beads.

**Figure 12:**
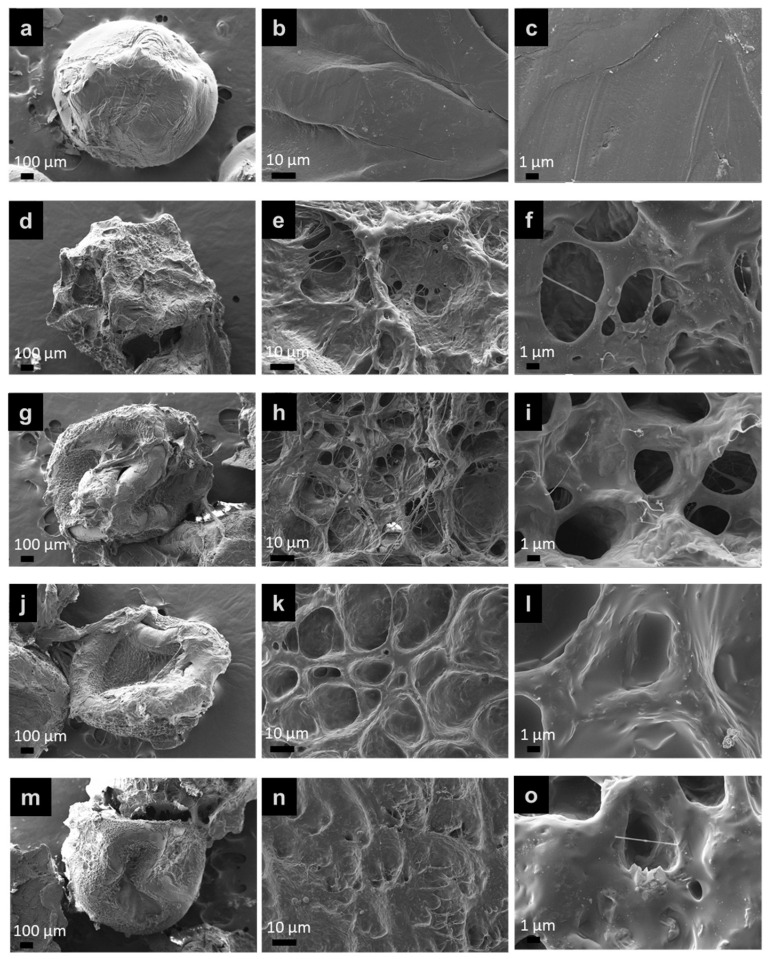
SEM images of (**a**–**c**) CS beads; (**d**–**f**) M0.50/P30-DPNR-coated CS beads; (**g**–**i**) M0.50/P30-DPNR/Si10-coated CS beads; (**j**–**l**) M0.50/P30-DPNR/Si20-coated CS beads and (**m**–**o**) M0.50/P30-DPNR/Si30-coated CS beads at ×50, ×1000 and ×10,000 magnifications.

The CS beads were prepared via the ionic gelation method. The different types of composites were used to coat onto CS beads. The physical appearance of crosslinked (PAA-*co*-PAM)-DPNR/silica-coated CS beads in the dry state is shown in Figure 11. All beads showed a spherical shape with a light-yellow color. Figure 12 shows the morphology of CS beads coated with different types of modified natural rubber/silica composites after immersion in water and freeze drying. The CS beads had a spherical shape with a size of about 1097.2 ± 149.3 µm. For the crosslinked (PAA-*co*-PAM)-DPNR/silica-coated CS beads with various silica contents, the size was higher than that of neat CS beads due to the coverage of high water-absorbing materials on CS beads. Their size was found to be in the range of 1246.2 ± 108.1 to 1398.9 ± 70.4 µm. Since the coating materials on CS beads exhibited high water absorption capacity, the porous morphology appeared on their surface. The pores on the surface of beads are formed via the sublimation of trapped water within the crosslinked network structure of the modified natural rubber/silica composite film after freeze drying [46]. The macro-pores formed on the surface of M0.50/P30-DPNR/CS beads with an average diameter of about 56.62 ± 8.12 µm were observed due to the access of water in their structure [47]. The interconnected porous structure was also found, and there were many small voids with a size of about 6.74 ± 2.96 µm on the wall of the samples. When the silica contents in the composites increased, it could be seen that the pore size tended to decrease. The average pore size was found to be 26.0 ± 5.6, 23.8 ± 5.1 and 18.2 ± 3.9 µm for the composites with 10, 20 and 30 phr of silica, respectively. The increase in the interaction of silica and modified natural rubber can reduce the spaces to adsorb water molecules [48]. These results corresponded to the decrease in the swelling degree when the silica was increased. Moreover, the pore wall thickness seemed to increase because the silica particles were buried in the wall of the composites. The porous framework became a closed-cell structure when the silica in the composites increased.

#### 3.3.2. Water Retention

**Figure 13:**
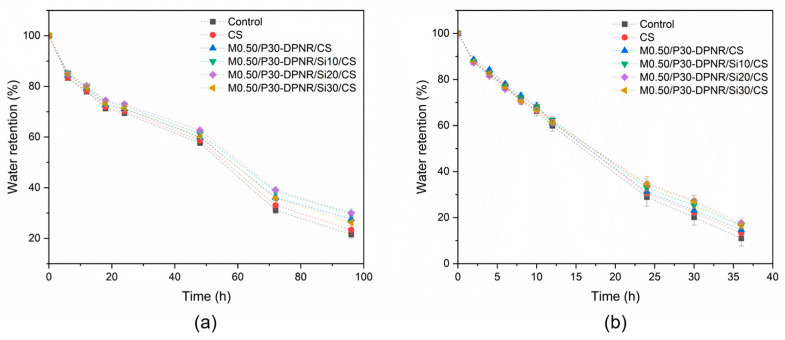
Water retention of different types of natural rubber/silica-coated CS beads at different temperatures: (**a**) 25 °C; (**b**) 45 °C.

The water retention abilities of samples with different temperatures at 25 and 45 °C were examined, as displayed in Figure 13. To compare the water retention ability of the samples, the different types of beads were buried in sand. The amount of water remaining during the period time was collected. In this study, the sand without beads was used as a control experiment. From the results, it was observed that the water retention decreased with storage time because of the loss in water in the sample holder from evaporation. The lowest values of water retention were observed in the case of the control experiment for all of the studied time periods at the temperature of both 25 and 45 °C. At 25 °C, the water retention value was 21.55 ± 1.48% for a control sample after 96 h. However, at 45 °C, its water retention value was only 10.99 ± 3.34% after 36 h because the evaporation rate of water increased at a high temperature. After burying the beads in sand, it was found that the existence of the beads resulted in an increase in water retention capacity. As can be seen from the results, the coated CS beads had better water retention capacity compared to the uncoated CS beads. It was suggested that the prepared coating materials had the ability to absorb water and were also effective materials for retaining water in their structure. It was also observed that the CS coated with M0.50/P30-DPNR/silica composites showed higher water retention than that of M0.50/P30-DPNR. The water retention increased with an increase in silica contents. For example, at 25 °C, the water retention of M0.50/P30-DPNR/Si10/CS and M0.50/P30-DPNR/Si20/CS was 29.64 ± 1.09 and 29.98 ± 1.81%, respectively, while the water retention of M0.50/P30-DPNR/CS was 27.67 ± 1.27% after 96 h. The water retention of M0.50/P30-DPNR/Si20/CS increased by 8.35 and 39.12% when compared to M0.50/P30-DPNR/CS and the control experiment at a temperature of 25 °C. Moreover, its water retention increased by 20.48 and 61.15% when compared to M0.50/P30-DPNR/CS and the control experiment at 45 °C. The increase in water retention capacity suggests that the presence of silica can improve mechanical stability to the composites because of the strong H-bonding interactions between the silica and polar groups of modified natural rubber [49]. However, the water retention of M0.50/P30-DPNR/Si30/CS was not much different from M0.50/P30-DPNR/Si20/CS. This is because the excess of silica loading leads to forming a silica–silica interaction, and the agglomeration of silica is obtained. This resulted in the decrease in its properties, including water adsorption and mechanical properties, as described in the above section. Therefore, it is noted that the modification of natural rubber with PAA-*co*-PAM can improve water adsorption, and the incorporation of silica can enhance the mechanical property of the composites for holding water in the structure. These would have advantages for reducing water consumption as these materials can maintain their structure with high water-absorbing and -retaining abilities [50].

#### 3.3.3. Loading Percentage and Release Behavior

**Figure 14:**
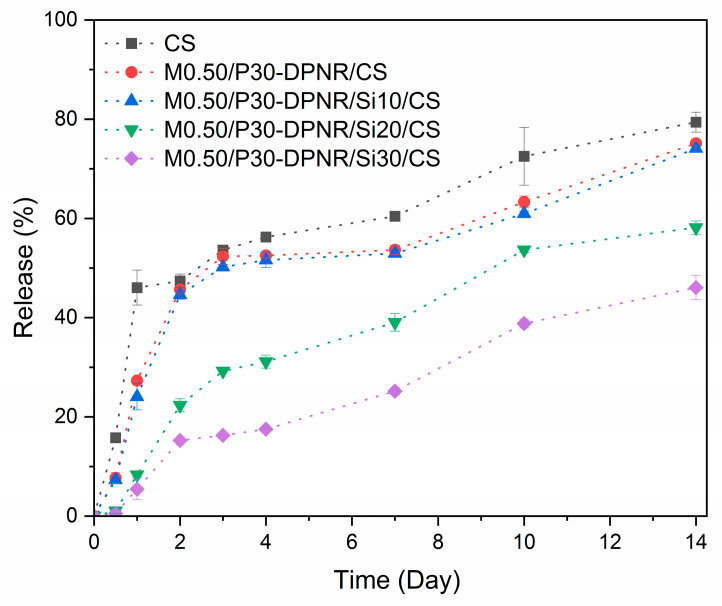
Release profiles of KNO_3_ from different types of natural rubber/silica-coated CS beads.

The loading capacity of the KNO_3_ in CS beads was determined. The KNO_3_ could be loaded in CS beads whereby the loading percentage was 40.55 ± 1.03%. This result was comparable to that reported by J.J. Perez [27]. Then, the KNO_3_-loaded CS beads were coated with various types of natural rubber/silica composites. The release behavior of KNO_3_ from the different types of beads in water (pH~6) was determined because this pH was in the range of the optimal pH (5.5 to 6.5) for the growth of most plants such as ginger, cassava, maize, wheat, French bean and tomato [51]. The fertilizer release profiles are shown in Figure 14. For comparison, the uncoated sample (CS) was also investigated. From the release profile, the released amount of KNO_3_ from CS reached 47.40 ± 1.37% after 2 days. Then, it reached 79.41 ± 2.01% after 14 days. The delayed release rate of KNO_3_ was observed when the beads were coated with the prepared natural-rubber-based composites. The release percentages of KNO_3_ from modified-natural-rubber/silica-coated CS beads were lower than those of the uncoated sample in all studied time periods. Thus, the coating with natural rubber/silica composites provided the controlled release of KNO_3_ in aqueous medium. The coating agent acted as a protective layer against the fast release of fertilizer [52]. After 2 days, the release percentages were found to be 45.68 ± 0.71, 44.61 ± 0.88, 22.32 ± 1.30 and 15.26 ± 0.56% for the CS beads coated with M0.50/P30-DPNR/silica composites with 0, 10, 20 and 30 phr, respectively. In order to study the release mechanism, the different kinetic models such as the zero-order kinetic model, first-order kinetic model, Higuchi model and Korsmeyer–Peppas model were applied to fit with the release data. The plots of various release kinetics are displayed in Figure S2 (see supplemental information) and the release kinetic parameters are presented in Table 4. The result shows that the release mechanism best fitted with the Korsmeyer–Peppas kinetic model with the highest value of the correlation coefficient (R^2^). According to this model, the equation is Q_t_ = kt^n^, where Q_t_ is the fraction of fertilizer release at time t, k is the release rate constant and n is the release exponent for indicating the characteristics of the release mechanism. For n < 0.5, the transport mechanism followed Fickian diffusion, whereby the diffusion is the main release mechanism. In the case of n > 1, the transport mechanism is classified as Super Case II transport, in which the nutrient transport mechanism is associated with the relaxation process of hydrophilic polymers upon swelling in water. From this result, 0.5 < n < 1 was obtained, indicating a non-Fickian transport release mechanism. This demonstrated that the release occurred through both processes [53]. The coating materials influence the retarding of the release and the diffusion of the encapsulated fertilizer through the coating layer. In this case, the higher amount of silica in the composites exhibited the lower release percentage of KNO_3_. The stronger interactions between the crosslinked (PAA-co-PAM)-DPNR and silica became more hydrophobic, a characteristic that provided the stronger barrier to prevent the release of KNO_3_ [54]. This resulted in a reduction in pore spaces in the network structure and a restriction to the access of water. From these characteristics, the KNO_3_ can be encapsulated in CS beads and the crosslinked (PAA-*co*-PAM)-DPNR/silica composites, as the outer layers allow the water and dissolved substances to pass through their network structure with a low diffusion rate. Therefore, these materials can retard the release and enhance the water holding capacity that would have potential for applications [55].

## 4. Conclusions

The crosslinked (PAA-*co*-PAM)-DPNR was successfully prepared via emulsion graft copolymerization in the presence of MBA as a crosslinking agent. The grafting efficiency and grafting percentage were increased from 38.9 ± 2.1 to 81.5 ± 3.5% and from 9.9 ± 1.7 to 24.4 ± 1.1% when MBA was increased from 0 to 0.50% w of the monomer, respectively. The addition of a crosslinking agent can hold the hydrophilic PAA-*co*-PAM chains to a natural-rubber-based material and form a network structure that has the ability of the absorption of water. The crosslinked (PAA-*co*-PAM)-DPNR was mixed with silica to prepare natural rubber/silica composites. Due to the strong interaction between polar groups of the crosslinked (PAA-*co*-PAM)-DPNR and silica, the composites became more hydrophobic, as determined via contact angle measurement. The swelling degree in the water of crosslinked (PAA-*co*-PAM)-DPNR/silica composites was found in the range of 2217.3 ± 182.0 to 8132.3 ± 483.8%, when the silica was added at 10 to 30 phr. The presence of silica was found to improve the mechanical properties of the composites. For the crosslinked (PAA-*co*-PAM)-DPNR incorporated with 20 phr of silica, its compressive strength and compressive modulus increased by 1.61 and 1.55 times those of the unloaded silica sample, respectively. The composites exhibited good structural stability without the breakage of samples after immersion in water and compression. These composites were employed as coating materials for fertilizer. The crosslinked (PAA-*co*-PAM)-DPNR/silica composites were coated on potassium-nitrate-loaded chitosan beads. They had the ability of retaining water molecules and exhibited slower potassium nitrate release. Therefore, these composites had the ability for water adsorption, water retention and controlled release behavior that would be useful for agricultural application.

## Figures and Tables

**Table 1:** Preparation of crosslinked (PAA-*co*-PAM)-DPNR/silica composites.

Ingredients	Contents (phr)			
	M0.50/P30-DPNR	M0.50/P30-DPNR /Si10	M0.50/P30-DPNR /Si20	M0.50/P30-DPNR /Si30
DPNR	100	100	100	100
Terric16A	5	5	5	5
CHP	1	1	1	1
TEPA	1	1	1	1
Acrylic acid	15	15	15	15
Acrylamide	15	15	15	15
MBA *	0.50	0.50	0.50	0.50
Silica	0	10	20	30

* Percentage by weight of monomer added.

**Table 2:** Thermal properties of M0.50/P30-DPNR/silica composites with various silica contents.

Samples	T_10_	T_max_	Residue at 600 °C
	(°C)	(°C)	(%)
MBA0.50/P30-DPNR	280.3	378.5	4.52
MBA0.50/P30-DPNR/Si10	281.5	381.5	10.47
MBA0.50/P30-DPNR/Si20	284.7	383.3	13.68
MBA0.50/P30-DPNR/Si30	289.0	384.2	26.03

**Table 3:** Compressive properties of M0.50/P30-DPNR/silica composites with various silica contents.

Samples	Compressive Strength at 80% Strain	Compressive Modulus at 5–10% Strain
	(MPa)	(MPa)
MBA0.50/P30-DPNR	10.71 ± 0.94	0.71 ± 0.13
MBA0.50/P30-DPNR/Si10	12.74 ± 0.93	0.84 ± 0.20
MBA0.50/P30-DPNR/Si20	17.29 ± 1.99	1.10 ± 0.13
MBA0.50/P30-DPNR/Si30	14.27 ± 1.64	0.98 ± 0.23

**Table 4:** Release parameters of release kinetic study.

Samples	R^2^				k (d^−1^)	n
	Zero-Order	First-Order	Higuchi	Korsmeyer–Peppas		
CS	0.6419	0.7126	0.7361	0.7541	30.64	0.5427
M0.50/P30-DPNR/CS	0.8218	0.8621	0.9098	0.9020	21.35	0.8096
M0.50/P30-DPNR/Si10/CS	0.8422	0.8776	0.9233	0.9239	20.08	0.8321
M0.50/P30-DPNR/Si20/CS	0.9298	0.9417	0.9647	0.9702	9.57	0.9519
M0.50/P30-DPNR/Si30/CS	0.8480	0.8541	0.9144	0.9437	5.54	0.9841

## Data Availability

Not applicable.

## Supplementary Materials

The following supporting information can be downloaded at https://www.mdpi.com/article/10.3390/polym15071770/s1: Figure S1: SEM image of silica particles; Figure S2: Release data fitted to different kinetic models: (a) zero-order, (b) first-order, (c) Higuchi model and (d) Korsmeyer–Peppas model.
