# Supplementary material for: Preparation of Crosslinked Poly(acrylic acid-co-acrylamide)-Grafted Deproteinized Natural Rubber/Silica Composites as Coating Materials for Controlled Release of Fertilizer
Source: Polymers (Basel). 2023 Apr 2;15(7):1770. doi: 10.3390/polym15071770 (PMC10097200; doi:10.3390/polym15071770)
Supplement: Supplementary file 1 [file polymers-15-01770-s001.zip › polymers-2278288-supplementary.pdf]

Supplementary Information

Preparation of Crosslinked Poly(acrylic acid-*co*-acrylamide)-*Grafted* Deproteinized Natural Rubber/Silica Composites as Coating Materials for Controlled Release of Fertilizer

Supharat Inphonlek [1,2], Kasama Jarukumjorn [1,2], Pranee Chumsamrong [1,2], Chaiwat Ruksakulpiwat [1,2,*] and Yupaporn Ruksakulpiwat [1,2,*]

[1] School of Polymer Engineering, Institute of Engineering, Suranaree University of Technology, Nakhon Ratchasima 30000, Thailand

[2] Research Center for Biocomposite Materials for Medical Industry and Agricultural and Food Industry, Suranaree University of Technology, Nakhon Ratchasima 30000, Thailand

* Correspondence: charuk@sut.ac.th (C.R.); yupa@sut.ac.th (Y.R.)

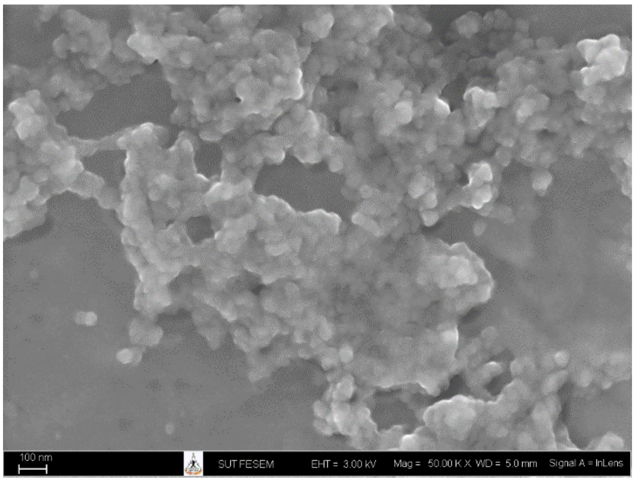

Figure S1. SEM image of silica particles.

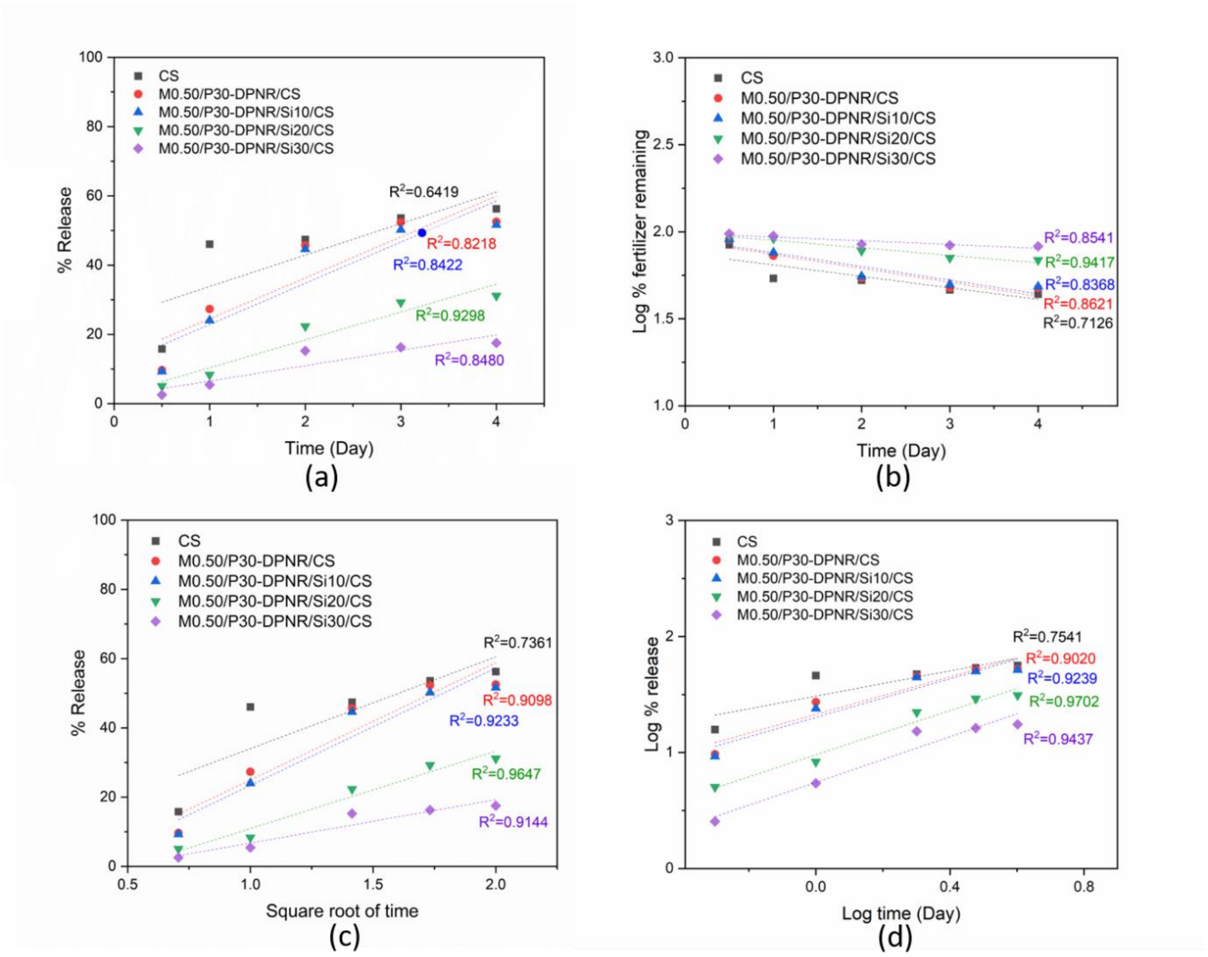

Figure S2. Release data fitted to different kinetic models; **(a)** zero-order, **(b)** first-order, **(c)** Higuchi model, and **(d)** Korsmeyer-Peppas models.
